# Mapping intestinal microenvironment heterogeneity in Hirschsprung's disease by spatial transcriptomics: Role of THBS1 in macrophage polarization and neural stem cell regulation

**DOI:** 10.1016/j.gendis.2026.102254

**Published:** 2026-05-26

**Authors:** Jinxin Li, Junbao Du, Lian Hou, Qing Luo, Quan Kang

**Affiliations:** a Department of General Surgery and Trauma, National Clinical Research Center for Child Health and Disorders, Ministry of Education Key Laboratory of Child Development and Disorders, Chongqing Key Laboratory of Structural Birth Defect and Reconstruction, Children's Hospital of Chongqing Medical University, Chongqing 400014, China; b Stem Cell Biology and Therapy Laboratory, National Clinical Research Center for Child Health and Disorders, Ministry of Education Key Laboratory of Child Development and Disorders, Chongqing Key Laboratory of Pediatrics, Children's Hospital of Chongqing Medical University, Chongqing 400014, China; c Stem Cell Biology and Therapy Laboratory, Children's Hospital of Chongqing Medical University, Chongqing 400014, China; d The First Affiliated Hospital, Zhejiang University School of Medicine, Hangzhou, Zhejiang 310002, China

**Keywords:** Hirschsprung's disease, Intestinal microenvironment, M2 macrophage, Spatial transcriptomics, THBS1

## Abstract

The pathogenic role of the intestinal microenvironment on Hirschsprung's disease (HSCR) is increasingly recognized; however, the spatial expression patterns and underlying molecular mechanisms of the HSCR intestinal microenvironment remain unclear. In this study, we generated the first comprehensive spatial transcriptomic map of HSCR and revealed microenvironmental heterogeneity. Spatial transcriptomics revealed THBS1 as a consistently down-regulated gene in HSCR aganglionic segments. *In vitro* experiments using THP-1 and SH-SY5Y cells showed that THBS1 promoted M2-like polarization and interacted with SDC1. Furthermore, THBS1-overexpressing macrophages enhanced the proliferation and migration of SH-SY5Y cells in co-culture. These findings suggest a potential role for THBS1 in modulating macrophage–neural interactions in the HSCR microenvironment, though further validation in primary cells is needed.

## Introduction

Hirschsprung's disease (HSCR) is a congenital malformation of the digestive tract that is common in newborns. It is characterized by the complete or partial absence of ganglion cells in the colon.^1^^,^^2^ This pathological defect results in persistent spasmodic contractions in the affected intestinal segment, leading to excessive intestinal motility and distension of the proximal colon.^3^^,^^4^ While surgical resection of the ganglion cell-deficient segment remains the primary treatment, some patients develop postoperative complications, such as fecal incontinence or constipation.^5^^,^^6^ Therefore, further investigation into the pathogenesis of HSCR and the development of novel therapeutic strategies are urgently required.

HSCR primarily results from aberrant migration, survival, and differentiation of enteric neural crest cells. Current research indicates that mutations in genes such as Ret, Gdnf, Gfra1, Nrtn, Ednrb, Et3, Zfhx1b, Phox2b, and Sox10 account for approximately 50% of known HSCR cases.^7^, ^8^, ^9^ Beyond genetic factors, recent studies have shifted focus to the “gut microenvironment theory”, which emphasizes the regulatory roles of the extracellular matrix (ECM) and soluble factors in enteric neural crest cell development, with microenvironmental abnormalities potentially contributing to disease pathogenesis.^10^, ^11^, ^12^ Accordingly, the role of the gut microenvironment in HSCR pathogenesis has gained increasing attention.

Developments in sequencing technologies have enhanced our understanding of the molecular biology of HSCR. Single-cell RNA sequencing has emerged as a cutting-edge technique for identifying, characterizing, and studying specific cell types and intercellular interactions.^13^ However, tissue dissociation into single-cell suspensions disrupts spatial information, causing gene expression profiles to lose their native tissue context and complicating the observation of authentic gene expression patterns. In this context, the emerging spatial transcriptomics (ST) technology, which leverages the spatial localization of gene expression, serves as an ideal complement to single-cell RNA sequencing.^14^^,^^15^ ST has been applied to non-small cell lung cancer and gastric cancer to verify the feasibility of spatial visualization and quantitative analysis of gene expression.^16^^,^^17^

In the present study, we performed ST sequencing of samples from patients with HSCR and healthy controls. By analyzing spatial gene expression patterns, we identified cells and key molecules potentially associated with disease mechanisms and explored the regulatory interactions between the gut microenvironment and disease progression. Our findings highlight the spatial heterogeneity of gene expression patterns and their clinical significance in the intestinal microenvironment, providing new insights into the pathogenesis and potential therapeutic strategies for HSCR.

## Materials and methods

### Human tissue and cell line

Intestinal tissues from the HSCR cohort were obtained from three independent infants (biological replicates) undergoing surgical resection for Hirschsprung's disease; control tissues were resected from one age-matched infant operated on for non-HSCR intestinal obstruction. From each HSCR patient, multiple segments (spastic, transitional, dilated, proximal) were collected as intra-patient regional samples. For ST, a total of eight tissue segments were processed: seven from the three HSCR patients (representing different intestinal regions) and one from the control patient (representing normal regions). The study protocol was approved by the Ethics Committee of Children's Hospital of Chongqing Medical University (Grant 2026 No. 76). Informed consent had been obtained from the parents or legal guardians of all participants. Fresh tissues for ST were embedded in optimal cutting temperature (OCT) compound and snap-frozen in isopentane pre-cooled with liquid nitrogen. Samples for Western blotting and quantitative real-time PCR were rapidly frozen and stored at −80 °C until use. Tissues utilized for immunohistochemistry analysis were formalin-fixed and paraffin-embedded. The human acute monocytic leukemia cell line THP-1 and the human neuroblastoma cell line SH-SY5Y were purchased from Procell Life Science & Technology (Wuhan, China).

### ST sequencing

Eight fresh intestinal segments were obtained through surgical resection, categorized into the dilated, transition, proximal, and spastic segments. The tissues were rinsed with pre-cooled phosphate-buffered saline solution and dried with gauze. Subsequently, the tissues were immersed in isoprene for freezing and transferred to cryopreservation tubes using forceps, followed by embedding in OCT mixture. The procedure was conducted as follows: i) Histological imaging was performed using hematoxylin–eosin staining; ii) Tissues underwent fixation, staining, and permeabilization to release mRNA, which then hybridized with probes containing a 16 bp dot-barcoding sequence and a 12 bp UMI sequence. Capture probes with poly(dT) sequences obtained gene expression information from the 3′-poly(A) tail by binding to mRNA; iii) cDNA synthesis and sequencing library preparation were performed using the captured RNA as a template; iv) Paired-end sequencing of the completed library was conducted on the 10 × Genomics Visium, as previously described.^18^

### Data processing and quality control

After sequencing, the data were visualized and analyzed using Space Ranger (version 1.1.0). The software integrated a reference genome database and performed data quality control with FastQC (Table S1). Additional sequencing was conducted, followed by data visualization using R software and other applications. The analysis utilized R software version 4.0.3 and the Seurat package version 3.1.1. After excluding low-quality units, the data were normalized using the SCTransform function to identify variable features and scale the data.

### Dimensionality reduction analysis

Principal component analysis was performed on the top 2000 most variable genes with the RunPCA function. The number of principal components indicated by the elbow plot was retained, and two-dimensional embeddings were generated with RunUMAP (perplexity = 30) (Table S2). Unsupervised clustering was subsequently performed with FindClusters at a resolution of 0.4 on the same principal components (Table S3). The resulting Uniform Manifold Approximation and Projection (UMAP) visualization revealed transcriptomic relatedness among spatial spots—neighboring spots exhibited highly similar expression profiles.

### Marker gene identification

The marker genes were identified using the likelihood-ratio test “bimod”, which selects genes up-regulated in each spot group compared with all other groups (Table S4). The screening criteria were: log fold-change threshold > 0 and minimum gene expression proportion across all cells > 0.25.

### Cell type identification

Cell type identification was performed using the “AddModuleScore” function in Seurat software. The method involved packaging the marker genes of the target cell type into a gene set and then calculating a “feature score” for each spot. Spots with higher scores were considered enriched with the target cell type. In addition, to estimate cell type composition within each 55 μm spot, we applied Robust Cell Type Decomposition (RCTD, version 2.0.0) for spot-level deconvolution. RCTD uses a supervised learning framework that leverages a reference single-cell RNA-sequencing dataset to decompose the mixed transcriptomic signal of each spot into estimated proportions of constituent cell types. RCTD was run in “multi” mode to allow for multiple cell types per spot. The reference dataset included eight major intestinal cell types: mesenchymal, epithelial, endothelial, myeloid, neuronal, T cells, B cells, and red blood cells. The deconvolution results were used to validate and refine the cluster annotations and to estimate cell type composition within each cluster across different tissue segments (Table S5–S12).

### Differential expression gene analysis

Differentially expressed genes were screened based on fold change and significance level (*p*-value), with Wilcoxon's signed-rank test as the default method (Table S13). Subsequently, Gene Ontology (GO) and Kyoto Encyclopedia of Genes and Genomes (KEGG) enrichment analyses were performed on the differentially expressed genes. The specific methodology involved using all protein-coding genes as a background list and the list of differentially expressed protein-coding genes as a candidate list screened from the background. The hypergeometric distribution test was conducted to calculate the *p*-values indicating whether the representative GO and KEGG functional sets were significantly enriched in the list of differentially expressed protein-coding genes (Table S14–S17). The *p*-values were then corrected for multiple testing using the Benjamini & Hochberg method to obtain *Q*-values. Additionally, the STRING database was utilized to identify interaction relationships among the differentially expressed genes, and a protein network interaction analysis diagram was constructed.

### Cellular communication analysis

To infer intercellular communication from ST data, we applied CellChat (version 1.6.0) to the normalized gene expression matrix of all spots. Given that each spot (55 μm diameter) may contain multiple cells, we adopted a spot-level approach as previously described for spatial data with mixed-cell spots. The approach briefly included the following steps:

**Data preprocessing:** The raw count matrix was normalized using the SCTransform function in Seurat (v4.0.3). For CellChat input, we used the normalized data without aggregation to single-cell resolution, as spatial spots were treated as composite “meta-cells”.

**Cell type annotation:** Each spot was assigned a primary cell type based on the maximum AddModuleScore from the cell type signature matrices (see *cell type identification*). Spots with scores below the 25th percentile for all cell types were classified as “mixed” and excluded from communication analysis to reduce noise.

**Interaction database:** We used the CellChatDB human database, which includes curated ligand–receptor interactions.

**Parameter selection:** Default parameters were used for most steps, with the following modifications to account for spatial data characteristics: i) population.size = TRUE to account for varying spot numbers per cluster; ii) truncatedMean = 0.1 to reduce the influence of outliers; iii) Significance was determined by permutation testing (*n* = 1000) with *p* < 0.05.

**Filtering strategy:** Only interactions where the ligand was expressed in at least 10% of spots in the sending cluster and the receptor in at least 10% of spots in the receiving cluster were considered. This threshold was chosen based on the median gene detection rate across spots.

**Addressing mixed-cell spots:** To mitigate the impact of spots containing multiple cell types, we performed sensitivity analysis by repeating the communication analysis after excluding spots with high heterogeneity scores (entropy > 0.8). The major ligand–receptor pairs (including THBS1–SDC1) were consistent with the full analysis, supporting the robustness of the findings.

The resulting communication networks were visualized using the netVisual_circle and netVisual_heatmap functions.

### Cell culture and transfection

THP-1 cells were selected as a model for macrophages due to their well-established use in studying human macrophage polarization and their responsiveness to THBS1 signaling. SH-SY5Y cells, a human neuroblastoma cell line, were used as a neuronal model. While SH-SY5Y cells are not enteric neural crest cells, they are derived from neural crest and retain many neuronal characteristics, including expression of neuronal markers, responsiveness to neurotrophic factors, and the ability to extend neurites. They are widely used as an *in vitro* model for studying neuronal differentiation, migration, and survival. However, we acknowledge that they are a cancer cell line and do not fully recapitulate the biology of primary enteric neurons or neural stem cells. Both THP-1 and SH-SY5Y cells were cultured in RPMI-1640 medium supplemented with 10% fetal bovine serum and 1% penicillin-streptomycin. For macrophage differentiation, THP-1 cells were treated with 100 nM phorbol 12-myristate 13-acetate (PMA, Sigma–Aldrich) for 48 h and then rested in fresh medium without PMA for 24 h before transfection. After 24 h of culture, THP-1 cells were transfected with 100 nM THBS1 siRNA (sequences provided below) using Lipofectamine 2000 (Invitrogen) in OptiMem (Gibco) medium. The cells were then returned to the growth medium for 6 h of continued culture. To achieve specific overexpression of THBS1, a plasmid was constructed. After 24 h of culture, THP-1 cells were transfected with 2 μg of the plasmid. The siRNA sequences targeting human THBS1 were: si-THBS1#1: sense 5′-GGA GAA ACU GAC UAC GAA UTT-3′, antisense 5′-AUU CGU AGU CAG UUU CUC CTT-3'; si-THBS1#2: sense 5′-CCA UCA CAA UGA CGA GUU ATT-3′, antisense 5′-UAA CUC GUC AUU GUG AUG GTT-3'; si-SDC1: sense 5′-GCA UGA GUG UGU CUA CUA UTT-3′, antisense 5′-AUA GUA GAC ACA CUC AUG CTT-3'. Non-targeting control siRNA (si-NC) was purchased from Nanjing Heyuan Biotechnology. The THBS1 overexpression plasmid (pCDNA3.1-THBS1) was verified by sequencing. Transfection efficiency was assessed by quantitative PCR and Western blotting 48 h post-transfection.

### RNA extraction and quantitative real-time PCR analysis

Total RNA was extracted from the cells and tissues using reagents (Hunan Aikorei Biological, China). After measuring the concentration with NanoDrop, RNA was reverse-transcribed into cDNA according to the instructions of the reverse transcription premixed kit (Hunan Aikorei Biological, China). The real-time quantitative PCR was performed using SYBR Green qPCR Master Mix (MCE, USA) on a Bio-Rad CFX96 instrument. The reaction conditions were: 95 °C for 30 s, followed by cycles of 95 °C for 5 s and 60 °C for 20 s, repeated 40 times to generate the melting curve. *Gapdh* was used as the internal reference gene, and the relative expression levels were calculated using the 2^–ΔΔCt^ method. The specific gene primers used in this study are listed in Table S18.

### Western blotting analysis

Tissue and cell samples were treated with RIPA buffer (Beyotime, P0013B) on ice and lysed by centrifugation at 15,000 rpm at 4 °C for 15 min. Equal volumes (20 μg) of protein lysates were separated by SDS-PAGE and detected using the BCA protein assay (Beyotime, P0010). The separated proteins were transferred to PVDF membranes (Millipore, USA). Membranes were blocked with 5% bovine serum albumin in Tris-buffered saline-Tween 20 at room temperature for 1 h and incubated at 4 °C overnight with primary antibodies at the following dilutions: anti-THBS1 (1:1000), anti-CD206 (1:1000), anti-ARG1(1:1000), anti-CD86 (1:1000), anti-SDC1 (1:500), anti-β-tubulin(1:5000), and anti-β-actin (1:5000). The membranes were then treated with a species-specific secondary antibody conjugated to horseradish peroxidase (HRP) (Epizyme Biomedical, LF102). Protein expression levels were visualized using chemiluminescent HRP developer (BIO-Rad, USA) on an iBright CL1500 analyzer.

### Immunohistochemistry

Tissue sections from all groups underwent dewaxing, hydration, and antigen retrieval in EDTA buffer. After blocking, the sections were incubated at 4 °C overnight with anti-CD206 antibody (1:200), followed by HRP-conjugated secondary antibody (1:500) at room temperature for 1 h. DAB staining was performed, and nuclei were hematoxylin-treated for 4 min. After dehydration and sealing, images were captured using a slide scanner (TEKSQRAY, SQS-40P).

### Immunofluorescence

THP-1 cell samples were collected from all groups, fixed with 4% paraformaldehyde for 15 min, permeabilized with 0.3% Triton X-100 for 10 min, and blocked with 5% bovine serum albumin for 30 min. The cells were incubated at 4 °C overnight with primary antibodies: anti-THBS1 (1:200), anti-CD206 (1:200), anti-CD86 (1:200), and anti-SDC1 (1:100). After washing, cells were incubated with Alexa Fluor 488- or 594-conjugated secondary antibodies (1:500, Invitrogen) at room temperature in the dark for 1 h. After counterstaining with DAPI for 5 min, the samples were mounted with antifade medium and imaged with a laser-scanning.

### Co-immunoprecipitation assay

A tatal of 1 × 10^7^ cells were collected and lysed in a RIPA buffer (Beyotime, P0013B) supplemented with protease and phosphatase inhibitors (MCE). Anti-THBS1 (Proteintech, 18304-1-AP), anti-SDC1 (Abclonal, A4174), or control IgG was added and the lysate was incubated at 4 °C overnight. Magnetic beads were then added and the mixture was rotated at room temperature for 1 h. After magnetic separation, the beads were washed three times with 0.5 mL lysis buffer, 50 μL loading buffer was added, and the mixture was heated at 95 °C for 5 min. The supernatant was collected and subjected to Western blotting analysis.

### Flow cytometry analysis

A tatal of 1 × 10^6^ THP-1 cells were collected from each treated group, washed with phosphate-buffered saline, and stained with fluorochrome-conjugated anti-CD206 (Proteintech, FITC-98031) and anti-CD86 (Proteintech, FITC-98025) according to the manufacturer's instructions. Flow cytometry was performed on a FACScan system (BD Biosciences), and the data were analyzed with FlowJo software. As shown in Figure S5, gating plots were added for the flow cytometry gating strategy.

### Transwell co-culture system

For non-contact co-culture experiments, THP-1 macrophages (after PMA differentiation and transfection) were seeded in the upper chamber of 6-well Transwell inserts (0.4 μm pore size, Corning, 3413) at 1 × 10^5^ cells per well, and SH-SY5Y cells were seeded in the lower chamber at 2 × 10^5^ cells per well. After 24 h of co-culture, SH-SY5Y cells were collected for functional assays (proliferation, migration, apoptosis). This system allows exchange of soluble factors but prevents direct cell–cell contact. Conditioned media experiments were not used for the data presented in Figure 6.

### CCK-8

The SH-SY5Y cells were removed from the chamber at 24, 48, 72, and 96 h, the supernatants were discarded, and 1 mL fresh medium and 100 μL CCK8 reagent were added to each well. The cells were incubated at 37 °C for 2 h, then the reaction mixture was transferred to a 96-well plate, and the absorbance at 450 nm was measured using an enzyme-labeled instrument.

### Clonal formation experiment

The SH-SY5Y cells were seeded at 1 × 10^3^/well in six-well plates with three replicates. The medium was changed every 4 days. After 2 weeks, the cells were fixed with 4% polyformaldehyde for 15 min, stained with crystal violet for 15 min, washed, dried, and then photographed to count the clones.

### Scarification test

The SH-SY5Y cells were cultured in the lower chamber of a 6-well plate until they reached 90% confluence, and then the cells were scraped vertically with a sterile pipette tip. The cells were washed with phosphate-buffered saline, and the healing rates were observed under a microscope at 0, 12, and 24 h.

### Transwell migration assay

The SH-SY5Y cells were seeded in the upper chamber of 8-μm Transwell chambers (Corning, USA), while the lower chamber was seeded with THP-1 cells transfected with siRNA or plasmid. After 24 h of incubation under standard conditions, the chambers were removed. The cells were fixed in 4% paraformaldehyde and stained with 0.1% crystal violet solution, followed by microscopic imaging.

### Cell apoptosis analysis

The SH-SY5Y cells in the co-culture chamber were collected with trypsin, rinsed with pre-cooled phosphate buffer, and resuspended in a binding buffer containing fluorescein isothiocyanate and propidium iodide (BestBio, China) labeled Annexin V. After incubation at 4 °C for 30 min, apoptotic cells were detected using FACScan (BD Biosciences).

### Statistical analysis

All statistical analyzes were performed using SPSS 18.0, with graphs created by GraphPad Prism 8.0. *P*-value < 0.05 was considered statistically significant.

## Results

### General profiles of ST sequencing in HSCR

To systematically investigate the spatial expression patterns of HSCR, we collected surgical specimens from three independent HSCR patients (biological replicates) and one non-HSCR control. From each HSCR patient, multiple intestinal segments (spastic, transitional, dilated, proximal) were obtained, yielding a total of eight samples for ST analysis. These specimens were categorized as spastic_1, dilated_1, proximal_1, control_1, transition_1, transition_2, dilated_2, and spastic_2. Freshly frozen samples were processed using ST sequencing on a 10x Genomics Visium platform, followed by histological staining, cDNA library preparation, and sequencing. Hematoxylin–eosin staining was used to delineate tissue boundaries and locations. Quantitative quality control using Space Ranger revealed that each sample contained 1939–3332 high-quality spots, with each spot of 55 μm in diameter. These spots represent mixed populations of cells potentially belonging to different cell types and were treated as individual units for further computational analysis and visualization. The average gene count per spot ranged from 1151 to 4421, which was consistent with the sequencing data.

To characterize global spatial organization, cluster analysis was performed on all spots and visualized using the UMAP method, revealing that all spots co-clustered into 18 clusters. To assign cell type identities to the 18 spatially defined clusters, we integrated three lines of evidence: i) histopathological annotation of tissue sections, ii) expression of canonical cell type marker genes, and iii) cell type proportion estimates from deconvolution (RCTD and Cell2location). Based on this integrated approach, the clusters were annotated as follows: Mucosal layer (Cluster 04, 05, 12, 15): High expression of EPCAM, KRT18, MUC2; RCTD estimated > 70% epithelial cells. Submucos (Cluster 01, 09, 16, 17): Enriched for COL1A1, DCN, VIM; RCTD estimated mixed fibroblast/smooth muscle populations. Lamina propria (Cluster 02, 03, 06, 07, 10, 13): Mixed populations including fibroblasts, immune cells, and smooth muscle; deconvolution showed variable proportions across clusters. Neural tissue (Cluster 14): High expression of neural markers (PRPH, S100B, ELFN1); RCTD estimated ∼40%–60% neuronal/glial cells, with the remainder being surrounding stromal cells (due to spot size mixing). Blood vessels (Cluster 11): Enriched for VWF, PECAM1, MCAM. Lymphoid aggregates (Cluster 08, Cluster 18): High expression of CD79A, MS4A1, CD3D; deconvolution confirmed > 50% B/T cells (Fig. 1A, D; Fig. S1). We next investigated the specific distribution of these 18 subgroups and their gene expression levels in the sample space sections (Fig. 1B). The results showed that Spastic1 and Spastic2 were absent in the neural tissue (Cluster 14). This aligns with the pathological findings in patients with congenital megacolon exhibiting the absence of ganglion cells in the interstitial and submucosal nerve plexuses. This demonstrated the reliability of the ST sequencing results. Besides, distinct spatial regions can be defined not only through histological evaluation but also through spatial gene expression patterns. We demonstrated the distribution of 18 clusters across 8 samples, revealing significant heterogeneity in the proportion of the same cluster across two different samples (Fig. 1C).

**Figure 1:**
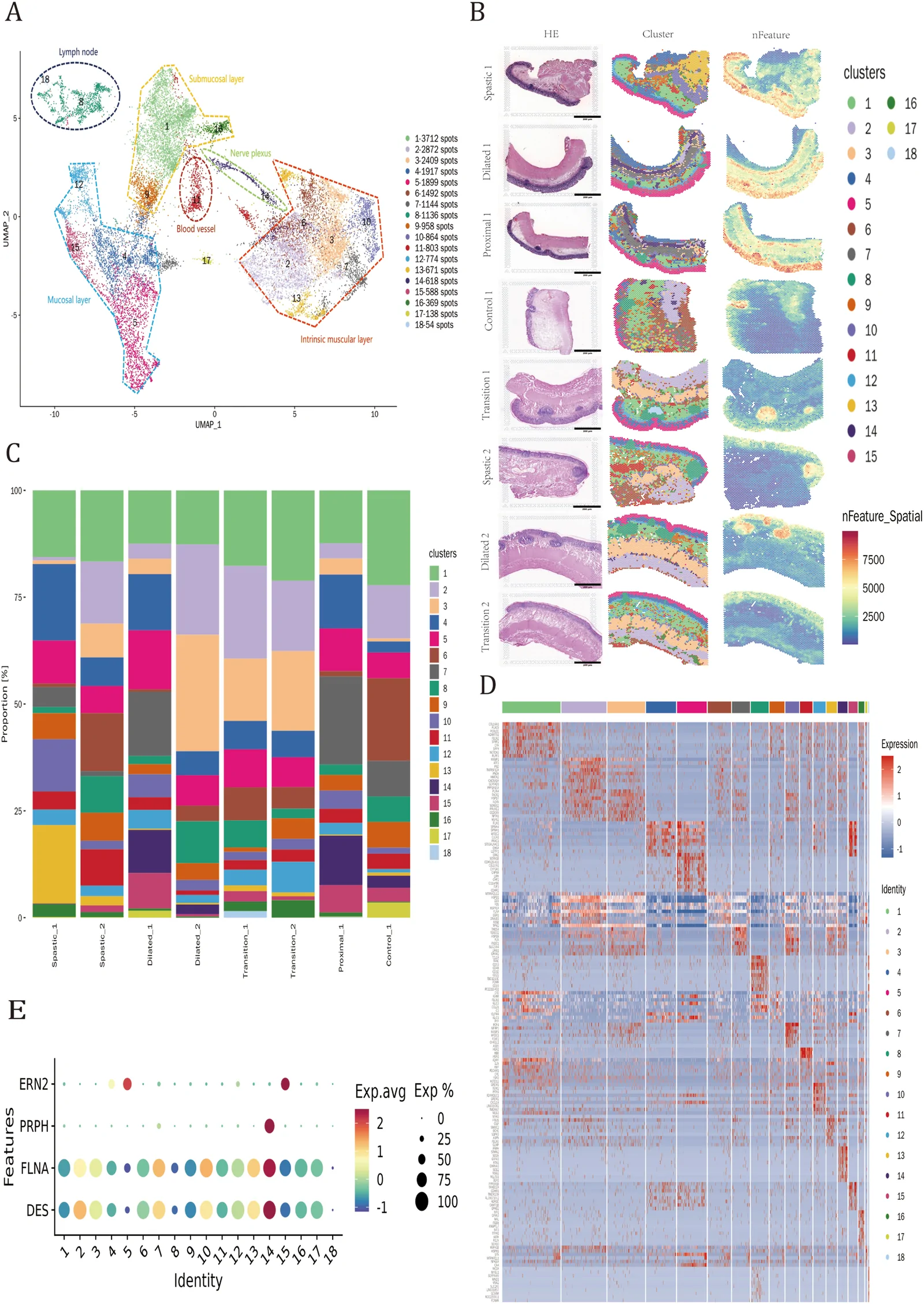
The spatial transcriptomic landscape of HSCR. **(A)** UMAP visualization of all points across 18 clusters (colored by cluster and spatial position). **(B)** Specific gene expression patterns and hematoxylin–eosin staining images for 18 clusters in sample space slices. Scale: 200 μm. **(C)** Proportional distribution of 18 clusters across different samples. **(D)** Gene expression profiles specific to 18 clusters. **(E)** Tissue-specific gene annotations across the eight tissues. Sample sizes: *n* = 3 HSCR patients (seven tissue segments) and *n* = 1 control (one segment).

To identify cellular phenotypic characteristics among different subpopulations, we performed a bimodal marker gene analysis across the 18 subpopulations, identifying genes that were specifically expressed within each subpopulation (Fig. 1D). To evaluate the sensitivity of the ST method for detecting point transcripts, we examined the spatial distribution of marker genes for common cell types in previously reported samples.^19^, ^20^, ^21^ The results confirmed that Des and Flna (markers typically associated with smooth muscle cells) were highly expressed in the intrinsic muscle layer, Prph (a marker typically associated with gastrointestinal neuronal cells) was localized in the neural tissue region, and Ern2 (a marker typically associated with gastrointestinal epithelial cells) was expressed in the mucosal layer. Together, these findings validate the reliability of the ST sequencing results (Fig. 1E).

Thus, our results preliminarily reveal significant differences in gene expression patterns among distinct spatial units (regions, clusters, and individual spots) within the HSCR intestinal microenvironment, indicating that these spatially resolved expression profiles play crucial roles in intestinal heterogeneity.

### Key gene atlas of ECM regulating enteric neural crest cells

Immune cells (monocytes, neutrophils, and M2 macrophages) and ECM components in the intestinal microenvironment play critical roles in the migration of enteric neural crest cells.^10^^,^^22^ We identified intestinal microenvironmental heterogeneity in HSCR. To further analyze the unique biological functions of each subgroup, we evaluated the expression of ECM- and immune cell-related genes across all clusters. The results revealed the following (Fig. 2A; Fig. S2A): Cluster 7, 10, 13, 14, 16, and 17 exhibited relatively high ECM scores; Cluster 5 and 8 exhibited relatively high monocyte scores; Cluster 5 and 18 exhibited relatively high neutrophil scores; and all clusters exhibited relatively high M2 polarization scores.

**Figure 2:**
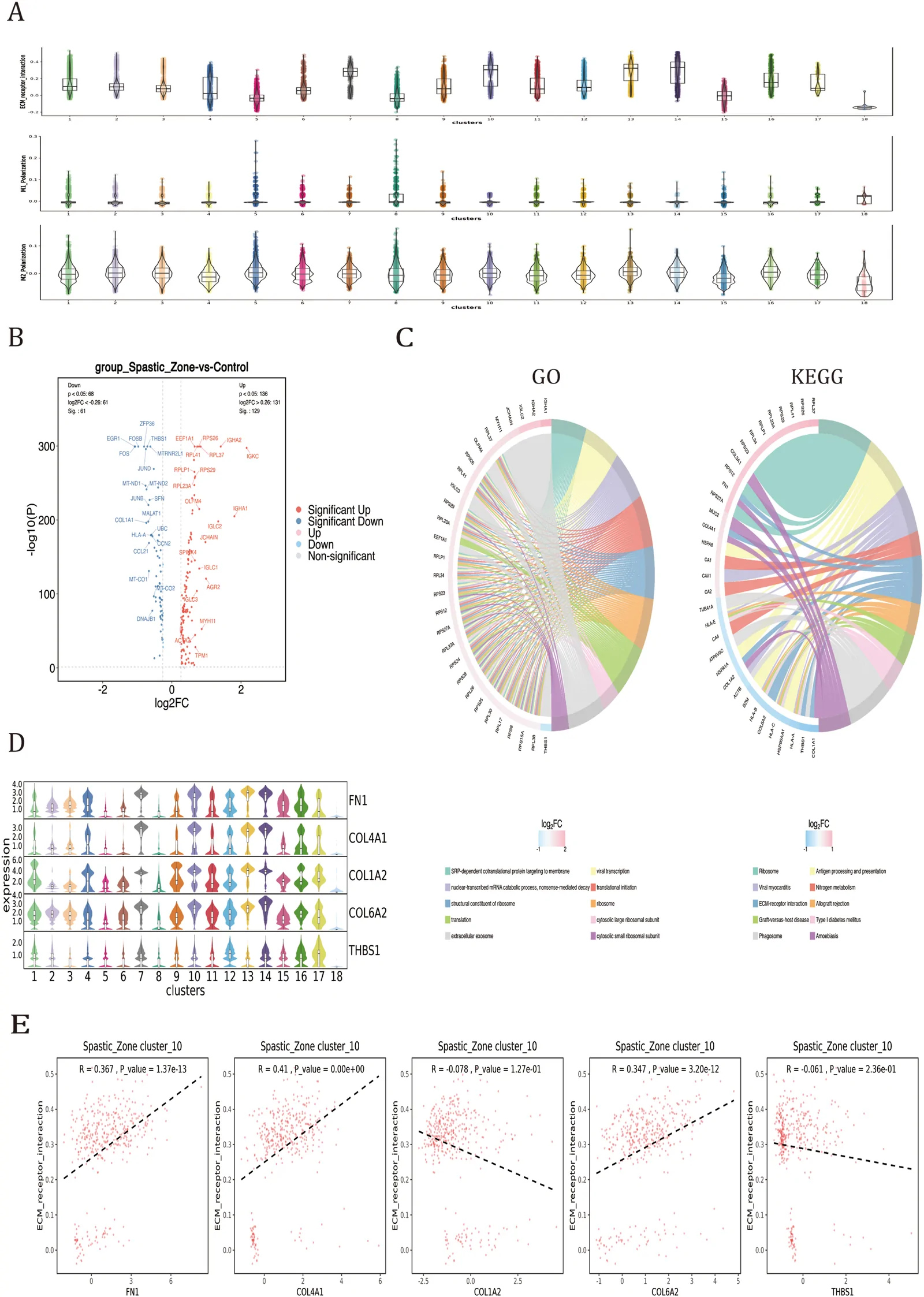
Key gene atlas of extracellular matrix (ECM) regulating the enteric neural crest cells. **(A)** Expression of ECM-and immune cell-related genes within each cluster. **(B)** Volcano plots of differentially expressed genes (DEGs) between the spastic and control groups. The DEGs were labeled. *p*-value < 0.05, Fold change > 1.2. **(C)** GO enrichment analysis and KEGG pathway analysis of the differentially expressed genes. **(D)** Violin plots of ECM-related genes in all clusters. The width of a violin plot indicates the kernel density of the expression values. **(E)** Correlation analyses on DEGs in the cluster with higher ECM scores. Sample sizes: spastic segments (*n* = 2), control segments (*n* = 1). Statistical significance for DEGs was determined by the Wilcoxon test with *p* < 0.05 and fold change > 1.2.

We noted that Figure 2A shows M2 macrophage scores in all clusters, including neural tissue (Cluster 14), which might appear inconsistent with the absence of neural tissue in spastic segments (Fig. 1B). This is explained by the resolution of ST: each 55 μm spot may contain multiple cell types. In Cluster 14 (neural tissue), deconvolution analysis estimated that neural cells constitute 40%–60% of the spot content, with the remainder being surrounding stromal and immune cells. Therefore, M2 macrophage signatures in these spots likely originate from the non-neural component of the mixed spot, rather than from macrophages residing within neural tissue. This underscores the importance of deconvolution for interpreting spatial data and the need for caution when assigning cell-type-specific signals to spots. However, because spot boundaries do not necessarily correspond to cell boundaries, the gene expression profiles of each spot may include multiple cells and/or partial cells rather than a single, discrete cell. Therefore, we selected the most heterogeneous spastic and control groups for differentially expressed gene analysis. In HSCR, most down-regulated genes (Egr1, Fosb, Junb, Thbs1, Col1a1, etc.) were key regulators in the inflammation-repair process, suggesting their involvement in enteric nervous system development (Fig. 2B).^23^ To explore the biological relevance of these transcriptional changes, we performed GO and KEGG functional enrichment analyses on the identified differentially expressed genes (Fig. 2C; Fig. S2B). Compared with the control group, the spastic group exhibited up-regulation of Fn1 and Col4a1 and down-regulation of Col1a2, Col6a2, and Thbs1 within ECM receptor interactions. Among these genes, Fn1, Col4a1, Col1a2, and Col6a2 are ECM-related protein-coding genes that influence intestinal neural crest migration through alterations in ECM components due to intestinal fibrosis.^24^ Using ST, we examined the distribution of these genes within clusters (Fig. 2D), with violin plots showing relatively high expression levels of Fn1, Col4a1, Col1a2, Col6a2, and Thbs1 in Cluster 10, 12, 13, 14, 16, and 17. Additionally, we performed correlation analyses between differentially expressed gene expression levels and ECM scores in subgroups with higher ECM scores (Fig. 2E; Fig. S2C). The results showed that the expression levels of Fn1, Col4a1, and Col6a2 were positively correlated with ECM scores, confirming their strong association with disease pathogenesis. In contrast, Thbs1, a gene responsible for encoding ECM proteins, was negatively correlated with ECM scores in the disease group, suggesting regulation of intestinal neural crest migration through alternative pathways.

Notably, while THBS1 expression was low in the fibrotic spastic segment, other pro-fibrotic genes such as Col4a1 and Fn1 were up-regulated (Fig. 2B and C), suggesting that fibrosis in HSCR may be driven by THBS1-independent mechanisms. This dissociation is further supported by the negative correlation between THBS1 expression and ECM score in the disease group (Fig. 2E). It should be noted that the M2 macrophage module scores were calculated based on gene expression signatures using AddModuleScore, which reflects the transcriptomic similarity to M2 macrophage markers rather than the actual count or proportion of M2 macrophages. RCTD deconvolution showed that myeloid cells constituted only 2%–15% of spots across clusters, indicating that the relatively high M2 module scores reflect gene expression patterns rather than abundant macrophage infiltration.

### Spatial expression pattern of Thbs1 in the intestinal microenvironment of HSCR reveals enrichment of M2 macrophages

Next, we screened cells potentially associated with the regulatory mechanism of THBS1 to investigate the underlying regulatory mechanisms linking the intestinal microenvironment to disease. Using the AddModuleScore method and standard cell-type marker gene sets, we assessed the enrichment levels of different cell types within each spot (Fig. 3A). The results showed that B cells were highly enriched in lymphoid nodules, Schwann cells in neural tissue regions, smooth muscle cells in the lamina propria, and epithelial cells in mucosal layers. These findings were consistent with the pathological observations. Furthermore, previous studies have reported that M2 macrophages and THBS1 exert positive effects on angiogenesis following myocardial infarction and that inhibition of THBS1 in renal research led to a reduction in M2 macrophages.^25^^,^^26^

**Figure 3:**
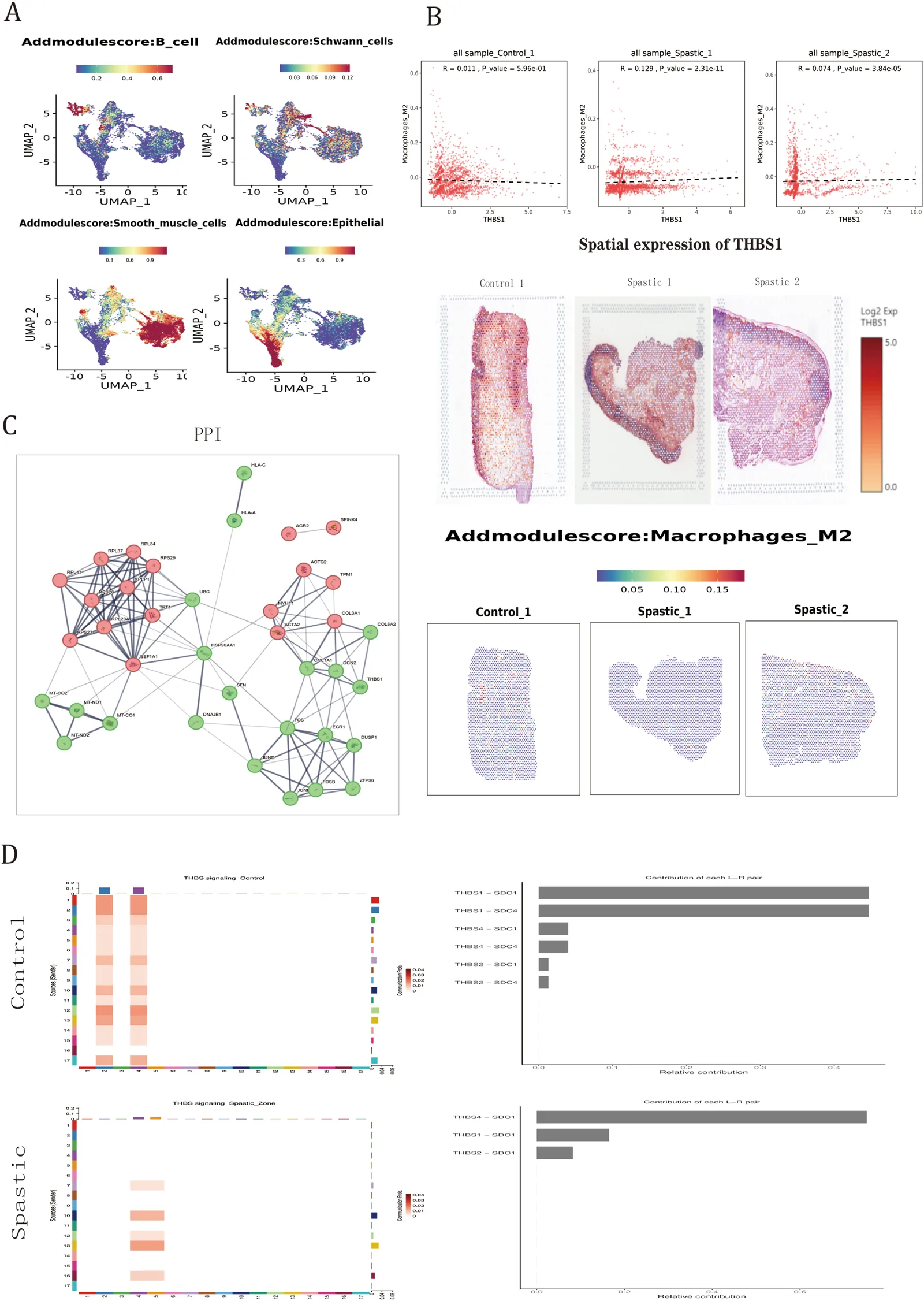
The spatial expression pattern of *Thbs1* in the HSCR intestinal microenvironment revealed M2 macrophage enrichment. **(A)** UMAP plot of enrichment fractions for various cell types. **(B)** Spatial expression and quantitative analysis of Thbs1 in M2 macrophages. **(C)** Protein network interaction analysis of differentially expressed genes. **(D)** Cell communication analysis of THBS1. Sample sizes: spastic segments (*n* = 2), control segments (*n* = 1). Correlation statistics: Pearson *r* and *p*-values are provided in the text.

To determine whether a similar relationship exists in HSCR, we compared Thbs1 expression with M2 macrophage abundance in the ST datasets (Fig. 3B). To quantitatively assess the spatial association between THBS1 expression and M2 macrophage abundance, we calculated Pearson correlation coefficients between THBS1 expression levels and M2 signature scores (based on CD163, MRC1, MSR1, and IL10) across all spots (Fig. 3B). A moderate positive correlation was observed in the spastic group samples (*r* = 0.129, *p* < 0.001), whereas the correlation in the control group was weak and statistically insignificant (*r* = 0.011, *p* = 0.596). These results suggest a potential spatial association that is disrupted in HSCR, but the correlation is modest and should be interpreted cautiously. Thbs1 and M2 signatures were low to moderately expressed and showed spatial overlap within the submucosa and lamina propria of spastic segments. To identify the interacting partners of Thbs1, we constructed a protein–protein interaction network based on differentially expressed genes (Fig. 3C; Fig. S3A). The results revealed that Thbs1 interacted with the ECM protein-coding genes Col6a2 and Col1a1, as well as the smooth muscle actin gene Acta2, suggesting a potentially significant role for Thbs1 in enteric neural crest cell migration. CellChat analysis further revealed that compared with the spastic group, Thbs1 and Sdc1 interactions were primarily observed in Cluster 2 and 4 in the control group (Fig. 3D; Fig. S3B). Furthermore, *in vitro* studies have shown that M2 macrophages can promote the proliferation and migration of SH-SY5Y cells while inhibiting their death and apoptosis.^27^ Thus, we hypothesize that during intestinal neural crest cell migration, THBS1 may facilitate the development of these cells by inducing M2 polarization of macrophages and that THBS1 interacts with SDC1.

Based on these multi-faceted observations—consistent down-regulation, negative correlation with ECM scores, spatial overlap with M2 signatures, and predicted interaction with SDC1—we selected THBS1 for further functional investigation as a candidate regulator of macrophage–neural interactions in HSCR.

### Expression of THBS1 and M2 macrophages in clinical samples

Quantitative real-time PCR revealed a significantly higher Thbs1 transcript abundance in control tissues than in HSCR tissues (*p* < 0.0001) (Fig. 4A). Western blotting and immunohistochemistry were performed to quantify THBS1, CD206 (M2 marker), and CD86 (M1 marker) expression. THBS1 and CD206 protein expression levels were elevated in control samples, whereas CD86 protein expression levels were reduced (*p* < 0.05) (Fig. 4B and C). Immunohistochemistry revealed cytoplasmic CD206 staining, which was consistently stronger in the control colons than in the HSCR colons (Fig. 4D). Together, these data indicate that both THBS1 and M2 macrophages were down-regulated in HSCR.

**Figure 4:**
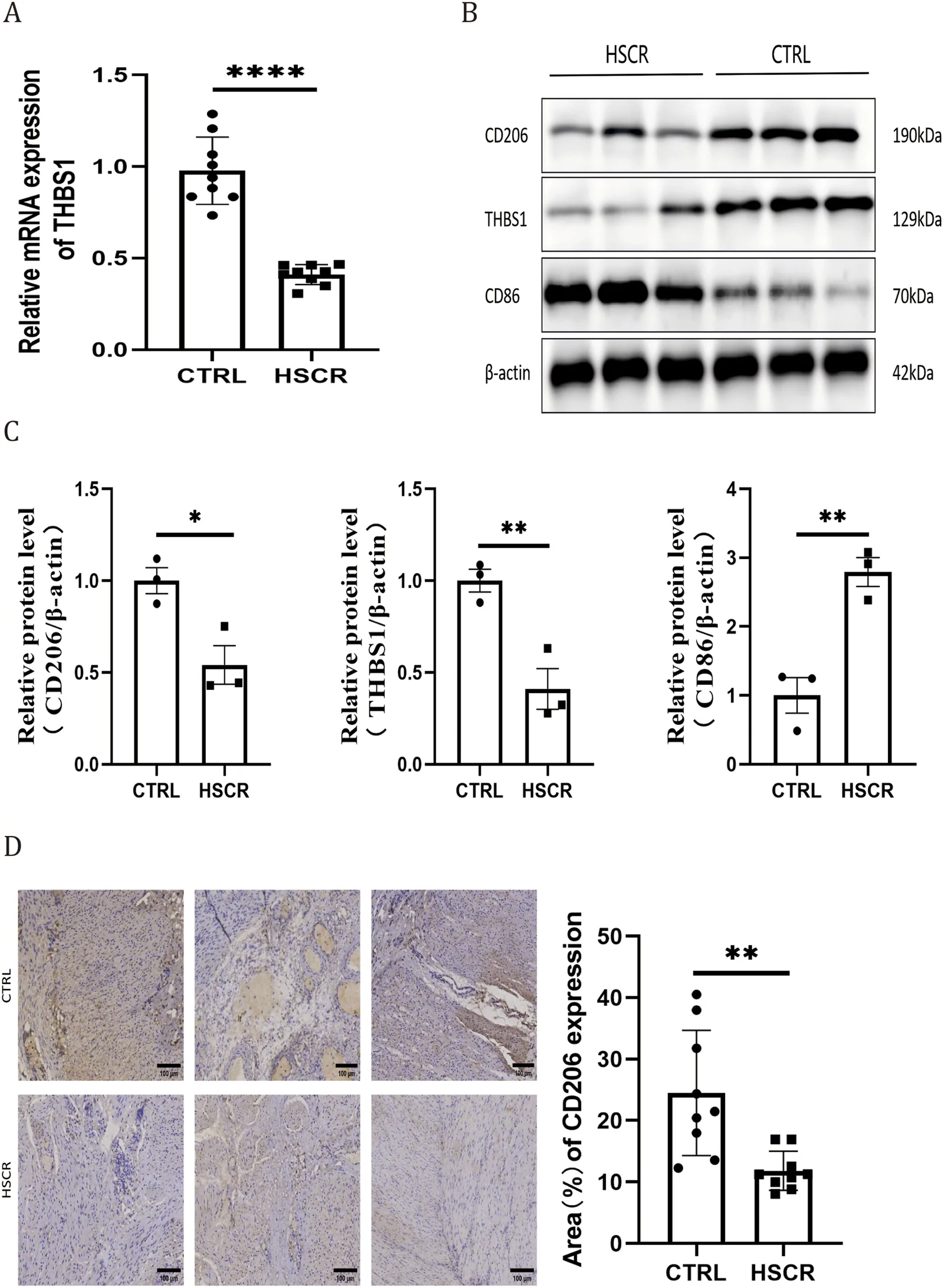
Validation of THBS1 and M2 macrophage expression in clinical samples. **(A)** Quantitative PCR analysis of *Thbs1* expression levels in clinical samples, with *Gapdh* as internal reference. **(B)** Western blotting analysis of THBS1, CD206, and CD86 protein expression levels in clinical samples, with β-Actin as internal reference. **(C)** Relative expression levels of THBS1, CD206, and CD86 in HSCR clinical specimens and controls. **(D)** Immunohistochemistry analysis of CD206 protein expression. Staining results: nuclei appear blue, while the target protein shows brownish-yellow positive staining. Sample sizes: HSCR (*n* = 3 patients), control (*n* = 3 patients). Data were expressed as mean ± standard deviation. Statistical significance was determined by an unpaired two-tailed Student's *t*-test. ∗*p* < 0.05, ∗∗*p* < 0.01, and ∗∗∗∗*p* < 0.0001. Scale: 100 μm.

### Effects of THBS1 on macrophage polarization

To investigate the effects of THBS1 on macrophage polarization, we used siRNA and plasmid transfection for Thbs1 knockdown and overexpression in THP-1 cells. Quantitative PCR and Western blotting results showed that compared with the negative control group, the knockdown group exhibited significantly decreased THBS1 expression at both the transcriptional and translational levels, whereas the overexpression group exhibited significantly increased THBS1 expression at these levels. Compared with the knockdown group, the recovery group exhibited increased THBS1 expression at both the transcriptional and translational levels (Fig. 5A–C). Subsequently, we analyzed the relative expression of the M1 marker CD86 and M2 markers CD206 and ARG1 using Western blotting. The data indicated increased CD86 expression in the knockdown group and increased CD206/ARG1 expression in the overexpression group (Fig. 5B and C; Fig. S4A). Next, we assessed macrophage polarization using flow cytometry. The experimental results showed increased proportions of CD86 in the knockdown group and increased CD206 proportions in the overexpression group (Fig. 5D; Fig. S4B). Additionally, cell immunofluorescence analysis of both marker genes revealed increased CD86 fluorescence intensity in the knockdown group and increased CD206 fluorescence intensity in the overexpression group (Fig. 5E; Fig. S4C). Increased CD86 and CD206 levels suggest that Thbs1 silencing and overexpression promoted M1-type and M2-type macrophage polarization, respectively. Our data indicate that macrophage M2 polarization was inhibited by Thbs1 silencing but enhanced by Thbs1 overexpression, suggesting that THBS1 influences macrophage polarization.

**Figure 5:**
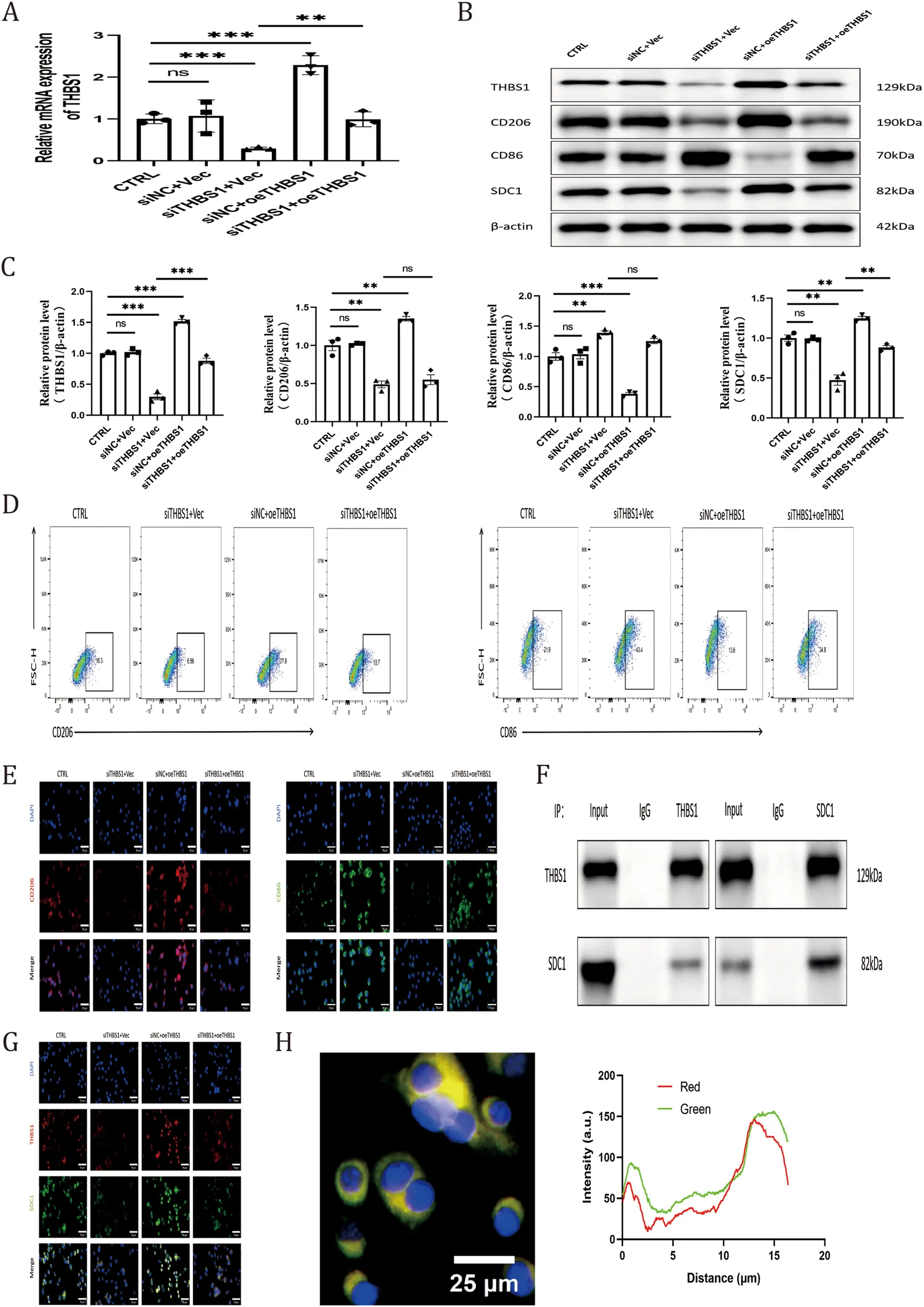
Effects of THBS1 on macrophage polarization. **(A)** Quantitative PCR analysis of *Thbs1* expression levels in THP-1 cells with knockdown and overexpression, using *Gapdh* as an internal reference. **(B)** Western blotting analysis of THBS1, CD206, CD86, and SDC1 protein expression in THP-1 cells, with β-Actin as internal reference. **(C)** Relative expression levels of THBS1, CD206, CD86, and SDC1 in THP-1 cells. **(D)** Flow cytometry analysis of M1 and M2 macrophage proportions in each group. **(E)** CD86 and CD206 immunofluorescence in THP-1 cells. Scale: 50 μm. **(F)** Immunoprecipitation assay confirming THBS1–SDC1 interaction. **(G)** THBS1–SDC1 immunofluorescence in THP-1 cells. Scale: 50 μm. **(H)** Spatial co-localization map of THBS1 and SDC1. Scale: 25 μm. All experiments were performed in triplicate (*n* = 3 independent biological replicates). Data were expressed as mean ± standard deviation. Statistical significance was determined by one-way ANOVA with Tukey's post-hoc test. ∗*p* < 0.05, ∗∗*p* < 0.01, ∗∗∗*p* < 0.001, and ∗∗∗∗*p* < 0.0001; ns, not significant.

Sequencing data revealed an interaction between THBS1 and SDC1. To further investigate the basis of this interaction in macrophages, we performed Western blotting to examine the relative expression of SDC1 (Fig. 5B and C). The results showed decreased SDC1 expression in the knockdown group and increased expression in the overexpression group. Compared with the knockdown group, the recovery group exhibited higher SDC1 expression. Subsequent co-immunoprecipitation experiments using total cell lysates and THBS1 antibodies confirmed the interaction between THBS1 and SDC1. Consistent with these findings, reverse co-immunoprecipitation experiments using total cell lysates and SDC1 antibodies validated this interaction (Fig. 5F). Dual-labeling immunofluorescence experiments were conducted to analyze the spatial distribution and dynamic changes in THBS1 and SDC1 cells (Fig. 5G and H; Fig. S4D). There is a decreased fluorescence intensity of SDC1 in the knockdown group and an increased fluorescence intensity in the overexpression group. THBS1 exhibited red fluorescence, whereas SDC1 exhibited green fluorescence. In THP-1 cells, both molecules were expressed in the nucleus and cytoplasm, with yellow regions indicating overlapping areas between THBS1 and SDC1 cells. Our data indicate that THBS1 and SDC1 interact with macrophages.

These results demonstrate that THBS1 modulates macrophage polarization *in vitro*, favoring an M2-like phenotype, and that THBS1 physically interacts with SDC1 in THP-1 cells. Whether this interaction directly mediates the polarization effect requires further investigation.

### THBS1 expression in macrophages influences SH-SY5Y cell proliferation, migration, and apoptosis

Previous studies have suggested that M2 macrophages exert positive regulatory effects on the biological behavior of SH-SY5Y cells.^28^ To validate the role of THBS1 expression in this process, we established a Transwell co-culture system in which THP-1 cells treated with Thbs1 via siRNA or plasmid transfection were placed in the upper chamber, and SH-SY5Y cells were placed in the lower chamber. First, cell viability was assessed using the CCK8 assay, revealing that macrophages in the overexpression group promoted SH-SY5Y survival, whereas those in the knockdown group inhibited survival (Fig. 6A). Colony formation assays further showed that overexpression in macrophages enhanced cell proliferation, whereas knockdown in macrophages suppressed it (Fig. 6B). Additionally, we assessed SH-SY5Y cell migration using Transwell and cell scratch assays. The results indicated that overexpression in macrophages exhibited enhanced migration capability, whereas knockdown in macrophages eliminated this effect (Fig. 6C and D). Finally, flow cytometry analysis of the apoptosis rate of SH-SY5Y cells revealed that the overexpression in macrophages inhibited apoptosis (Fig. 6E). Collectively, these co-culture experiments indicate that THBS1-overexpressing macrophages can modulate the behavior of SH-SY5Y cells *in vitro*, promoting their proliferation and migration while reducing apoptosis. Whether these effects translate to enteric neural crest cells *in vivo* remains to be determined.

**Figure 6:**
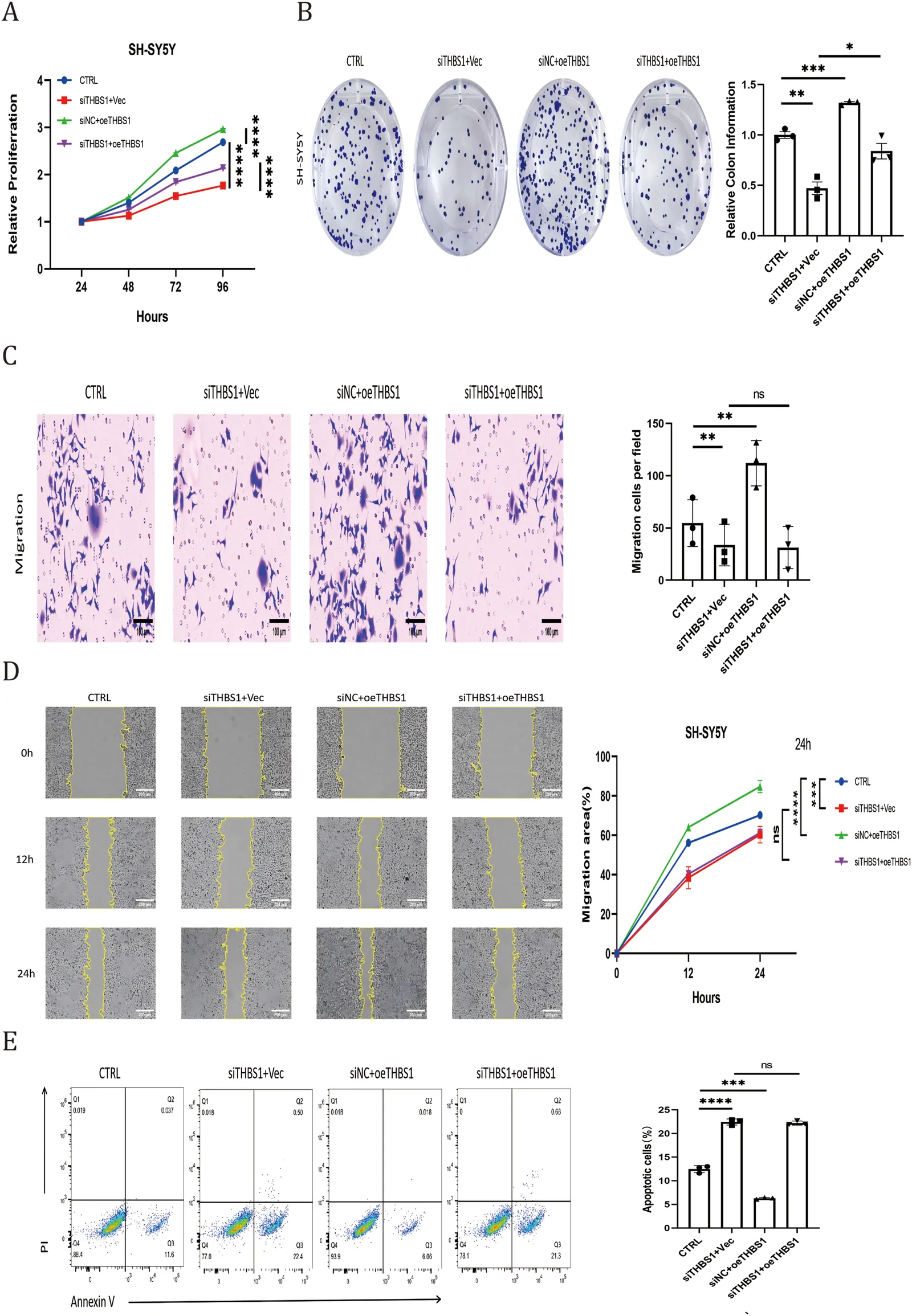
Expression of THBS1 in macrophages affects the proliferation, migration, and apoptosis of SH-SY5Y cells. **(A)** CCK8 assay for SH-SY5Y viability. **(B)** Colony-forming assay for SH-SY5Y proliferation. **(C)** Transwell assay for SH-SY5Y migration. Scale: 100 μm. **(D)** Cell scratch assay for SH-SY5Y migration. Scale: 200 μm. **(E)** Flow cytometry for SH-SY5Y apoptosis rate. ns: not statistically significant; ∗*p* < 0.05, ∗∗*p* < 0.01, ∗∗∗*p* < 0.001, and ∗∗∗∗*p* < 0.0001. Data shown are from non-contact Transwell co-culture experiments (0.4 μm pore size) as described in the methods. All experiments were performed in triplicate (*n* = 3 independent biological replicates). Data were expressed as mean ± standard deviation. Groups: CTRL (untreated control), siTHBS1+Vec (THBS1 knockdown with empty vector), siNC + oeTHBS1 (control siRNA with THBS1 overexpression), siTHBS1+oeTHBS1 (THBS1 knockdown with THBS1 overexpression, rescue group). Note that the rescue group showed partial but incomplete restoration of the phenotypes compared with the siNC + oeTHBS1 group, particularly in migration assays. Statistical significance was determined by one-way ANOVA with Tukey's post-hoc test. ∗*p* < 0.05, ∗∗*p* < 0.01, ∗∗∗*p* < 0.001, and ∗∗∗∗*p* < 0.0001; ns, not significant.

We also evaluated the rescue group (siTHBS1+oeTHBS1) to determine whether re-expression of THBS1 could reverse the effects of THBS1 knockdown. In all assays, the rescue group partially restored the phenotypes compared with the knockdown group, but did not fully reach the levels of the overexpression group (siNC + oeTHBS1) (Fig. 6A–E). This incomplete rescue suggests that additional factors may contribute to the observed effects, or that the efficiency of THBS1 re-expression was not sufficient to completely counteract the knockdown. Notably, the incomplete restoration was most pronounced in the migration assays (Fig. 6C and D), indicating that THBS1 may cooperate with other macrophage-derived signals to fully modulate neuronal cell behavior.

## Discussion

In this study, we applied ST to map the intestinal microenvironment of HSCR and identified THBS1 as a consistently down-regulated gene in aganglionic segments. The main findings of this exploratory study are: i) hierarchical clustering and dimensionality reduction analysis classified all spots into 18 subpopulations and established their spatial relationships; ii) marker gene analysis revealed distinct cellular phenotypic characteristics among these subpopulations; iii) key cells and molecules were screened through differential gene expression analysis, GO/KEGG enrichment analysis, CellChat profiling, ECM scoring, and spatial expression patterns of immune cells; iv) THBS1 expression and M2 macrophage abundance were reduced in HSCR; v) *in vitro*, THBS1 was found to promote M2-like polarization of THP-1 macrophages and to interact with SDC1; and vi) in co-culture experiments, THBS1-overexpressing macrophages enhanced SH-SY5Y cell proliferation and migration while reducing apoptosis. These observations point to a potential modulatory role for THBS1 in the intestinal microenvironment of HSCR, though direct evidence in primary human cells and *in vivo* models is still lacking.

Single-cell RNA sequencing provides a high-throughput, high-resolution transcriptomic profiling of individual cells, enhancing our understanding of cellular state diversity and heterogeneity.^29^ However, it requires cell isolation, which may disrupt the spatial context of the tissues. The physical location of cells is crucial for understanding tissue functions and related pathological changes. ST is an *in situ* RNA sequencing technology that captures mRNA while preserving spatial tissue information.^30^ Whole-tissue transcriptome sequencing allows for comparisons across different regions of the same tissue. HSCR is a complex disease arising from interactions between genetic and environmental factors.^31^, ^32^, ^33^

ST enabled us to preserve tissue architecture while profiling gene expression across HSCR intestinal segments. This approach revealed not only the expected absence of neural clusters in aganglionic regions but also a distinct spatial pattern of THBS1 expression and its proximity to M2 macrophage signatures. These observations align with emerging evidence that the gut microenvironment, particularly immune cells, may influence enteric nervous system development. However, as noted in the results, the spatial resolution of ST (55 μm spots) does not allow single-cell assignment, and the correlation between THBS1 expression and M2 signatures does not imply causation. Thus, these spatial findings should be viewed as hypothesis-generating rather than definitive.

We acknowledge that the sample size for ST is modest. Given the exploratory nature of this study and the technical complexity and cost of ST, we prioritized depth of spatial resolution per sample over cohort size. To assess the robustness of THBS1 down-regulation, we performed permutation-based testing: THBS1 expression was compared between spastic and control spots across 1000 random subsamples of spots, and the down-regulation was consistently observed (empirical *p* < 0.01). Furthermore, all key findings from ST were validated by orthogonal methods (quantitative PCR, immunohistochemistry) in independent tissue sections from the same patients, providing additional confidence in the results.

THBS1 is a matricellular protein with diverse functions, including modulation of cell adhesion, migration, and immune responses.^34^^,^^35^ Previous studies have shown that THBS1 can influence monocyte chemotaxis and wound healing.^36^^,^^37^ In our study, we observed that THBS1 overexpression in THP-1 cells was associated with increased expression of the M2 marker CD206, suggesting a potential link between THBS1 and macrophage polarization. We also detected a physical interaction between THBS1 and SDC1 in these cells. However, the relationship between THBS1 and M2 macrophage polarization remains poorly understood. Additionally, our study revealed that its binding to SDC1 plays a dominant role in the THBS1 signaling pathway. Previous studies have suggested that down-regulation of SDC-1 might inhibit the progression of cellular fibrosis by regulating the TGF-β1/THBS1 signaling pathway.^34^^,^^38^ Intestinal fibrosis is thought to contribute to HSCR development,^24^ indicating that the THBS1–SDC1 receptor–ligand pair plays a role in HSCR pathogenesis. Furthermore, differential protein network interaction analysis suggested an association between THBS1 and ECM protein-coding genes. Based on our *in vitro* findings, it is speculated that the THBS1-SDC1 interaction may contribute to macrophage polarization.

An apparent paradox in our study is the down-regulation of THBS1 in the spastic, fibrotic segment of HSCR, given its well-established pro-fibrotic functions in other tissues such as the liver, kidney, and lung. In those contexts, THBS1 activates latent TGF-β and promotes ECM deposition. However, THBS1 is a matricellular protein with context-dependent roles. During enteric nervous system development, THBS1 may primarily function as a guidance cue for migrating neural crest cells and as a modulator of immune cell polarization, rather than as a fibrotic mediator. Indeed, THBS1 has been shown to support neuronal migration and axonal growth in other developmental systems. The fibrotic changes observed in HSCR may arise from distinct mechanisms—such as chronic inflammation, mechanical obstruction, or activation of alternative pro-fibrotic pathways (*e.g.*, up-regulation of Col4a1, Fn1, as seen in our data)—that override or bypass THBS1-dependent pathways. Future studies should dissect the spatiotemporal dynamics of THBS1 function in intestinal fibrosis versus neurogenesis using lineage-specific and *in vivo* models.

The incomplete rescue observed in our co-culture experiments after THBS1 re-expression in THBS1-knockdown macrophages (Fig. 6) suggests that THBS1 may not be the sole mediator of macrophage-induced effects on neuronal cells. It is possible that THBS1 knockdown also affects other macrophage functions or that compensatory pathways are activated during the knockdown period, which are not fully reversed by subsequent THBS1 re-expression. Alternatively, the timing or level of THBS1 re-expression in our rescue experiments may not have been optimal. These observations highlight the complexity of macrophage–neural crosstalk and warrant further investigation into additional factors that might cooperate with THBS1 in this context.

HSCR primarily results from the aberrant migration, survival, and differentiation of enteric neural crest cells. Studies have shown a significant interactive dialog and developmental interdependence between macrophages and enteric neurons.^39^^,^^40^ To further investigate the regulatory role of muscle macrophages in enteric neuronal development, we conducted *in vitro* cytological experiments to examine the effects of macrophages on the biological characteristics of SH-SY5Y cells. Co-culturing THBS1-transfected macrophages with SH-SY5Y cells revealed that THBS1 overexpression promoted neural stem cell proliferation and migration and inhibited apoptosis. However, the mechanisms by which macrophages regulate neural stem cell differentiation remain to be elucidated.

We recognize that the use of THP-1-derived macrophages and SH-SY5Y neuroblastoma cells does not fully recapitulate the enteric neural crest cell biology central to HSCR. SH-SY5Y cells, while commonly used as a neuronal model, are of sympathetic adrenal origin and do not perfectly mimic enteric neurons. Similarly, THP-1 cells are a monocytic cell line and may not reflect the full phenotypic spectrum of tissue-resident macrophages. Nevertheless, these models provide a tractable system for initial mechanistic exploration. Future studies should validate our findings using primary enteric neural crest cells and intestinal macrophages, or using human induced pluripotent stem cell-derived enteric neurons and macrophages in co-culture systems. Such approaches will be essential to confirm the translational relevance of the THBS1–SDC1–macrophage axis in HSCR.

There are some limitations in this study. First, these findings should be interpreted with caution due to the small sample size. Second, the control tissues were obtained from infants with intestinal obstruction rather than from healthy individuals. While we selected macroscopically normal segments proximal to the obstruction and confirmed the absence of histological inflammation, obstruction-related changes such as mild ischemia, altered motility, or subclinical inflammation may have influenced the baseline gene expression profile. Third, using THP-1 and SH-SY5Y cell lines cannot fully recapitulate the biology of primary human intestinal macrophages and enteric neurons. THP-1 cells are a monocytic leukemia cell line and may not reflect the full phenotypic spectrum of tissue-resident macrophages, which are shaped by their local microenvironment. Although SH-SY5Y cells are derived from the neural crest and share some characteristics with neural crest-derived cells, they are not equivalent to primary enteric neural crest cells or neural stem cells. They exhibit cancer-associated alterations in signaling pathways and may not accurately model normal developmental processes such as migration, differentiation, and survival.

In summary, this study employed ST to comprehensively map the heterogeneity of the intestinal microenvironment in HSCR, establishing a spatial gene expression atlas. Our ST analysis revealed down-regulation of THBS1 in HSCR aganglionic segments and its spatial association with M2 macrophage signatures. *In vitro* experiments using cell lines suggested that THBS1 can promote M2-like polarization and interact with SDC1, and that THBS1-overexpressing macrophages can modulate neuronal cell behavior in co-culture.

## CRediT authorship contribution statement

**Jinxin Li:** Writing – original draft. **Junbao Du:** Data curation. **Lian Hou:** Investigation. **Qing Luo:** Supervision, Resources. **Quan Kang:** Writing – review & editing, Supervision.

## Data availability

All raw sequencing data from this study have been uploaded to the Genome Sequence Archive (GSA) of the National Genomics Data Center (NGDC) (accession number: HRA017074). The raw data have been deposited in the GSA database under the link https://ngdc.cncb.ac.cn/gsa-human/s/A7zVE8mC, and the processed data and analysis code have been deposited in the GitHub repository under the link https://github.com/zerozero7356/Spatial-transcriptomics-about-HSCR.

## Funding

This research was supported by the Chongqing Natural Science Foundation (China) (No. TC2020JCYJ-MSXMX0113).

## Conflict of interests

The authors declared no competing interests.
