# Supplementary material for: Mapping intestinal microenvironment heterogeneity in Hirschsprung's disease by spatial transcriptomics: Role of THBS1 in macrophage polarization and neural stem cell regulation
Source: Genes Dis. 2026 May 26;14(2):102254. doi: 10.1016/j.gendis.2026.102254 (PMC13694789; doi:10.1016/j.gendis.2026.102254)
Supplement: Supplementary file 1 [file mmc1.docx]

Supplementary Materials

**Mapping intestinal microenvironment heterogeneity in Hirschsprung’s disease by spatial transcriptomics: role of THBS1 in macrophage polarization and neural stem cell regulation**

Jinxin Li, Junbao Du,Lian Hou , Qing Luo, Quan Kang

**Supplementary Tables**

**Table S1.**Statitics for QC

**Table S2.**UMAP reduced dimensional data

**Table S3.**Clusters information

**Table S4.**top10 markers for each cluster

**Table S5.**The mean cell type proportions for each cluster in Control_1 sample

**Table S6.**The mean cell type proportions for each cluster in Dilated_1 sample

**Table S7.**The mean cell type proportions for each cluster in Dilated_2 sample

**Table S8.**The mean cell type proportions for each cluster in Proximal_1 sample

**Table S9.**The mean cell type proportions for each cluster in Spastic_1 sample

**Table S10.**The mean cell type proportions for each cluster in Spastic_2 sample

**Table S11.**The mean cell type proportions for each cluster in Transition_1 sample

**Table S12.**The mean cell type proportions for each cluster in Transition_2 sample

**Table S13.**Differentially expressed genes between group spastic and control

**Table S14.**GO background

**Table S15.**KEGG background

**Table S16.**GO enrichment analysis between group spastic and control

**Table S17.**KEGG enrichment analysis between group spastic and control

**Table S18.** Primers used in qPCR

**Supplementary Figures and Figure Legends**

**Figure. S1**

**Figure. S1** The mean cell type proportions for each cluster in each sample.

**Figure. S2**

**Figure. S2** **Key Gene Atlas of ECM Regulating the ENCCs.**

**A.**Expression of neutrophils and monocytes in each cluster.**B.**GO and KEGG functional enrichment analysis of differentially expressed genes between spastic group and control group.**C.** ECM correlation of differentially expressed genes in clusters 13 and 16.

**Figure. S3**

**Figure. S3 A.**Protein network interaction analysis of differentially expressed genes.**B.**Differential interaction strength network

**Figure. S4**

**Figure. S4 A.**Western blot analysis of ARG1 protein expression in THP-1 cells, with β-Tubulin as internal reference.**B.**Flow cytometry analysis of M1 and M2 macrophage proportions in each group. **C.**CD86 and CD206 immunofluorescence in THP-1 cells.**D.**THBS1-SDC1 immunofluorescence in THP-1 cells.

**Figure. S5**

**Figure. S5 A.**Flow cytometry gating strategy for CD86.**B.**Flow cytometry gating strategy for CD206.**C.**Flow cytometry gating strategy for apoptosis.
